# Thermodynamic Irreversibility Analysis of Thermal Radiation in Coal-Fired Furnace: Effect of Coal Ash Deposits

**DOI:** 10.3390/ma16020799

**Published:** 2023-01-13

**Authors:** Chong Zhang, Zhongnong Zhang, Chun Lou

**Affiliations:** 1 State Key Laboratory of Coal Combustion, School of Energy and Power Engineering, Huazhong University of Science and Technology, Wuhan 430074, China; 2 Yantai Longyuan Power Technology Co., Ltd., Yantai 264006, China

**Keywords:** thermal radiation, thermodynamic irreversibility analysis, radiative entropy generation, coal ash deposits

## Abstract

In this paper, a three-dimensional (3-D) high-temperature furnace filled with a gas-solid medium was investigated, and the radiative transfer equation and the radiative entropy transfer equation in the chamber were applied in order to analyze the effect of coal deposits on thermal radiation. The heat flux on the walls of the furnace and the entropy generation rate were determined due to the irreversibility of the radiative heat transfer process in the furnace. Furthermore, the effect of ash deposits on the wall surface on the irreversibility of the radiation heat transfer process was investigated. The numerical results show that when burning bituminous and sub-bituminous coal, ash deposits in the furnace led to a 48.2% and 63.2% decrease in wall radiative heat flux and a 9.1% and 12.4% decrease in the radiative entropy rate, respectively. The ash deposits also led to an increase in the entropy generation number and a decrease in the thermodynamic efficiency of the radiative heat transfer process in the furnace.

## 1. Introduction

In order to mitigate carbon emission caused by coal combustion and achieve the aim of carbon neutrality, it is necessary to improve the efficiency of coal-fired boiler furnaces and reduce coal consumption [1,2,3]. According to the second law of thermodynamics, the thermodynamic irreversibility of the heat transfer process in a boiler furnace leads to a certain loss of available work, and it will further affect the efficiency of coal-fired boiler furnaces. [4,5]. Considering that thermal radiation is the main mode of heat transfer in coal-fired furnaces, it is of great interests to investigate thermodynamic irreversibility due to radiative heat transfer in high-temperature coal-fired furnaces [6].

Entropy generation is an important parameter in the thermodynamic analysis, and it is associated with thermodynamic irreversibility. So, theoretical analysis of radiative entropy generation (REG) is the basis for the analysis of irreversibility in high-temperature furnaces. The conduction and validation of REG are based on the establishment of a radiative entropy transfer model [7,8,9]. Considering a high-temperature system filled with radiative medium and surrounded by an opaque solid wall, the REG is found from the irreversibility of the absorption, emission and scattering processes of the medium [7] and the radiative heat transfer on the wall [8]. Based on the numerical solution of REG, the effects of medium temperature and radiative properties of medium and wall on REG in a one-dimensional (1-D) high-temperature system furnace with different boundary conditions were investigated numerically [10,11,12].

Besides the above-mentioned investigation, Makhanlall [13] derived the REG in a gray gas-particle two-phase medium and conducted validation through the Gouy–Stodola theorem. It is obvious that this analytical method is better suited to the practical industrial furnace situation. Therefore, this approach was applied to various problems of thermodynamic analysis of high-temperature systems including 1-D high-temperature systems with participating medium including CO_2_, H_2_O, even soot [14,15], lab-scale hydrocarbon diffusion flames [16,17,18], as well as practical combustion facilities [19,20,21]. During the above studies, it was found that the thermodynamic role of thermal radiation in the combusting flows should be paid more attention, and the variation in temperature and radiative properties in combustion chambers would have crucial effects on REG.

In the actual operation of coal-fired boiler furnaces, ash that arises even in complete combustion inevitably forms deposits on the wall and heat exchanger surfaces of the furnace due to mineral and slag formation during combustion [22]. These ash deposits decrease the boiler performance by being the leading obstacles to heat transfer [23,24,25]. Specifically, the ash deposits influence thermal radiation significantly because they alter wall emissivity [26,27]. From the point of view of the second law of thermodynamics, the ash deposits will have a significant effect on the REG and thermodynamic efficiency of radiative heat transfer in a furnace. The evaluation of the effect of ash deposits on REG is of great importance for the operation and design of a furnace. However, few works have focused on REG analysis of thermal radiation in coal-fired furnaces caused by coal ash deposits. Only Zhang and Lou [28] preliminarily analyzed the impact of wall spectral emissivity on the spectral characteristics of heat flux and REG in a high-temperature three-dimensional (3-D) enclosure, in which wall spectral emissivity was set as 1 in special spectral ranges but 0.2 in other wavelengths. Actually, the radiative properties (emissivity and reflectivity) of coal ash deposits can be determined by measurement of the radiation emitted or reflected from the object at a known temperature [29,30,31]. Furthermore, Baxter et al. [30] developed a method for implementing in situ measurements of ash deposit emissivity. The measurements were performed using an apparatus consisting of a heating device, optics, and a Fourier transform infrared (FTIR) emission spectrometer. Moore et al. [31] also used the method to measure the spectral emittance of deposits left after burning bituminous and sub-bituminous coals in situ. Additionally, some measurement results for the thermoluminescence properties of gamma irradiated clinker and Portland cement were analyzed [32,33,34]. The results showed that the gamma intensity depends on the dopants and their concentration, and it decreases with the increase in the thickness of the considered shields.

In general, the emissivity of coal ash deposits has been measured, and the numerical model of the REG has been proposed. However, to the best of the author’s knowledge, no work has been reported to evaluate the effect of ash deposits on REG. In this paper, a thermodynamic analysis model in a 3-D coal-fired furnace was established. Among the radiation media considered, solid particles due to incomplete combustion of pulverized coal were considered for the first time. Then, the heat flux and REG in the furnace were obtained based on the measured wall emissivity with ash deposits. Moreover, the effect of wall spectral emissivity on the spectral characteristics of heat flux and entropy generation were analyzed.

## 2. Numerical Models and Formulation

### 2.1. Formula for REG Rate

The 3-D coal-fired furnace considered in this paper was assumed to mainly contain non-gray gases (CO_2_ and H_2_O) and particles (soot, char and ash particle), and to be surrounded by non-gray walls. According to previous studies of REG analysis [8,9,13,14,15], the total REG is caused by the irreversibilities of three processes. They are: (1) the absorption and emission processes of CO_2_, H_2_O, soot, char and ash particles; (2) the scattering process of char and fly ash; and (3) the absorption, emission, and reflection processes on the wall.

The local REG rates due to the absorption and emission processes of CO_2_, H_2_O, soot, char and fly ash particle are $S_{ae,{\mathrm{CO}}_{2}}^{\prime \prime \prime }$, $S_{ae,H_{2}O}^{\prime \prime \prime }$, $S_{ae,\mathrm{soot}}^{\prime \prime \prime }$, and $S_{ae,p}^{\prime \prime \prime }$, respectively. Hence, the local REG rate due to the absorption and emission processes of medium $S_{ae}^{\prime \prime \prime }$ equals the sum of $S_{ae,{\mathrm{CO}}_{2}}^{\prime \prime \prime }$, $S_{ae,H_{2}O}^{\prime \prime \prime }$, $S_{ae,\mathrm{soot}}^{\prime \prime \prime }$, and $S_{ae,p}^{\prime \prime \prime }$, which are as given in [12,14]:

(1) $$\begin{array}{l} S_{ae,{\mathrm{CO}}_{2}}^{\prime \prime \prime }\left(r\right)=-\sum_{\mathrm{bands}}\int_{\Omega }\sum_{i=1}^{N_{q}}\sum_{j=1}^{N_{q}}\omega_{i}\omega_{j}k_{g_{i},\Delta \eta ,{\mathrm{CO}}_{2}}\left[I_{b,\eta }\left(r\right)-I_{\Delta \eta ,ij}\left(r,s\right)\right]\left[\frac{1}{T\left(r\right)}-\frac{1}{T_{\eta ,ij}\left(r,s\right)}\right]d\Omega \Delta \eta ,\\ S_{ae,H_{2}O}^{\prime \prime \prime }\left(r\right)=-\sum_{\mathrm{bands}}\int_{\Omega }\sum_{i=1}^{N_{q}}\sum_{j=1}^{N_{q}}\omega_{i}\omega_{j}k_{g_{j},\Delta \eta ,H_{2}O}\left[I_{b,\eta }\left(r\right)-I_{\Delta \eta ,ij}\left(r,s\right)\right]\left[\frac{1}{T\left(r\right)}-\frac{1}{T_{\eta ,ij}\left(r,s\right)}\right]d\Omega \Delta \eta ,\\ S_{ae,\mathrm{soot}}^{\prime \prime \prime }\left(r\right)=-\sum_{\mathrm{bands}}\int_{\Omega }\sum_{i=1}^{N_{q}}\sum_{j=1}^{N_{q}}\omega_{i}\omega_{j}k_{\eta ,\mathrm{soot}}\left[I_{b,\eta }\left(r\right)-I_{\Delta \eta ,ij}\left(r,s\right)\right]\left[\frac{1}{T\left(r\right)}-\frac{1}{T_{\eta ,ij}\left(r,s\right)}\right]d\Omega \Delta \eta ,\\ S_{ae,p}^{\prime \prime \prime }\left(r\right)=-\sum_{\mathrm{bands}}\int_{\Omega }\sum_{i=1}^{N_{q}}\sum_{j=1}^{N_{q}}\omega_{i}\omega_{j}k_{\eta ,p}\left[I_{b,\eta }\left(r\right)-I_{\Delta \eta ,ij}\left(r,s\right)\right]\left[\frac{1}{T\left(r\right)}-\frac{1}{T_{\eta ,ij}\left(r,s\right)}\right]d\Omega \Delta \eta , \end{array}$$

where T is the temperature of the gas and particles; $I_{b,\eta }$ is the spectral blackbody radiative intensity at temperature T; $T_{\eta ,ij}$ is the spectral radiative temperature, which can be obtained through substituting the blackbody intensity by an arbitrary monochromatic intensity [7]; $I_{\Delta \eta ,ij}$ is the spectral radiative intensity, which can be obtained by solving the radiative transfer equation (RTE); $k_{g_{i},\Delta \eta ,{\mathrm{CO}}_{2}}$, $k_{g_{j},\Delta \eta ,H_{2}O}$, $k_{\eta ,\mathrm{soot}}$ and $k_{\eta ,p}$ are the spectral absorption coefficients of the CO_2_, H_2_O, soot, char and ash particles, respectively; $\omega_{i}$ and $\omega_{j}$ are the weights of the *i*th and *j*th quadrature points; and $N_{q}$ is the number of quadrature points.

The local REG due to the scattering process of char and ash particles $S_{s}^{\prime \prime \prime }$ is as given in [8]:

(2) $$S_{s,p}^{\prime \prime \prime }\left(r\right)=\sum_{\mathrm{bands}}\int_{\Omega }\sum_{i=1}^{N_{q}}\sum_{j=1}^{N_{q}}\omega_{i}\omega_{j}\sigma_{\eta ,p}\left(\frac{1}{4\pi }\int_{\Omega^{\prime }}\frac{I_{\Delta \eta }\left(r,s^{\prime }\right)}{T_{\eta }\left(r,s^{\prime }\right)}\Phi \left(s,s^{\prime }\right)d\Omega^{\prime }-\frac{I_{\Delta \eta }\left(r,s\right)}{T_{\eta }\left(r,s\right)}\right)d\Omega \Delta \eta ,$$

where $\sigma_{\eta ,p}$ is the spectral scattering coefficient of char and ash particles.

The local REG rate due to the absorption, emission, and reflection processes on the wall $S_{W}^{\prime \prime }$ is given as in [8,9]:

(3) $$S_{W}^{\prime \prime }\left(r_{W}\right)=\sum_{\mathrm{bands}}\int_{\Omega }\sum_{i=1}^{N_{q}}\sum_{j=1}^{N_{q}}\omega_{i}\omega_{j}\left[\frac{I_{\Delta \eta ,ij}\left(r_{W},s\right)}{T_{W}}-L_{\eta }\left(r_{W},s\right)\right]\left(n_{W},s\right)d\Omega \Delta \eta ,$$

where $L_{\eta }$ is the spectral radiative entropy intensity, and $T_{W}$ is the wall temperature.

The total REG rate can be defined by the sum of the local terms as:

(4) $$S_{G}=\int_{V}S_{ae}^{\prime \prime \prime }\left(r\right)+S_{s}^{\prime \prime \prime }\left(r\right)dV+\int_{A}S_{W}^{\prime \prime \prime }\left(r_{W}\right)dA.$$

Furthermore, based on the calculation of the total REG rate, the entropy generation number (EGN) characterizing the degree of irreversibility of the radiative heat transfer process is as given in [9]:

(5) $$M=\frac{S_{G}\cdot T_{0}}{Q},$$

where $T_{0}$ is the ambient temperature, which is set as 300 K.

### 2.2. Solution of Radiative Heat Transfer

From Equations (1)–(3), it can be seen that the RTE for the 3-D furnace should be solved to obtain the spectral radiative intensity. The temperatures of gases and particles in the furnace are the same at thermal equilibrium. Considering the non-gray spectral radiative properties of gases and particles, the RTE for a narrow-band Δη can be calculated as:

(6) $$\begin{array}{ll} \frac{dI_{ij,\Delta \eta }\left(r,s\right)}{ds} & =\left(k_{g_{i},\Delta \eta ,{\mathrm{CO}}_{2}}+k_{g_{j},\Delta \eta ,H_{2}O}+k_{\eta ,\mathrm{soot}}+k_{\eta ,p}\right)I_{b,\eta }\left(r\right)\\ & -\left(k_{g_{i},\Delta \eta ,{\mathrm{CO}}_{2}}+k_{g_{j},\Delta \eta ,H_{2}O}+k_{\eta ,\mathrm{soot}}+k_{\eta ,p}\right)I_{g_{i}\Delta \eta }\left(r,s\right)\\ & +\frac{\sigma_{\eta ,p}}{4\pi }\int_{4\pi }\Phi \left(s,s^{\prime }\right)I_{g_{i}\Delta \eta }\left(r,s^{\prime }\right)ds^{\prime }, \end{array}$$

where $I_{g_{i}\Delta \eta }$ is the corresponding radiative intensity under the distribution function value *g* in the narrow-band Δη; $I_{b,\eta }$ is the spectral radiative intensity of the black body under the central wavenumber in the narrow band Δη; $k_{\eta ,\mathrm{soot}}$, $k_{\eta ,p}$ and $\sigma_{\eta ,p}$ can be obtained by Rayleigh’s theory and Mie theory with the known volume fractions of soot, char and ash particles, respectively [35].

The statistical narrow band correlated-k (SNBCK) model was used to obtain the spectral absorption coefficients of CO_2_ and H_2_O [36]. In the SNBCK model, the total spectrum is divided into multiple narrow bands. In each narrow band, the spectral radiative intensity of a blackbody does not vary with wavenumber. The actual absorption coefficient distributions of gases can be expressed by the *f* distribution and the *g* distribution. Since the *g* distribution function increases monotonically, the calculation of average radiative intensity can be simplified via using a Gauss–Lobatto quadrature scheme. Furthermore, the average spectral radiative intensity in the narrow band Δη can be calculated as:

(7) $${\bar{I}}_{\Delta \eta }=\sum_{i=1}^{N_{q}}\sum_{j=1}^{N_{q}}\omega_{i}\omega_{j}I_{ij,\Delta \eta }.$$

Additionally, the local radiative heat flux density *q* and total heat flux *Q* on the wall can be calculated as follows:

(8) $$q\left(r_{W}\right)=\sum_{\mathrm{bands}}\int_{4\pi }{\bar{I}}_{\Delta \eta }\left(r_{W}\right)\left(n_{W}\cdot s\right)d\Omega \Delta \eta ,$$

(9) $$Q=\int_{W}q\left(r_{W}\right)dA.$$

## 3. Numerical Procedure

In addition, considering the coal ash deposits on the wall surface, the spectral emittance of deposits used in this paper came from the in situ measured results by Moore et al. [31] using FTIR. These parameters were the input values to solve the RTE and obtain the REG.

### 3.1. Simplified Combustion Model

The simplified combustion model was established to evaluate gas radiation models for radiative heat transfer in a 3-D real-size virtual coal-fired furnace by Kez et al. [31]. The coal-fired furnace is approximated by a rectangular enclosure with the dimensions of *L_x_* × *L_y_* × *L_z_* = 20 m × 20 m × 50 m, in which the temperature and concentrations can be prescribed through the analysis of thermodynamic state of the unburned coal-oxidizer mixture and given as [35]:

(10) $$\Phi \left(r\right)=f_{r}\left(r\right)\Phi_{W}+\left(1-f_{r}\left(r\right)\right)\Phi_{C},$$

where Φ is the physical quantity including the temperature and mole fractions of CO_2_ and H_2_O, $r={\left[{\left(x-L_{x}/2\right)}^{2}+{\left(y-L_{y}/2\right)}^{2}\right]}^{0.5}$ is the distance to center-line, $f_{r}$ is the radial factor, and the subscripts *W* and *C* represent the positions along the wall and the center-line, respectively. A third-order polynomial function of $f_{r}\left(r\right)={\left(\min\left(\frac{2r}{L_{x}},1\right)\right)}^{3}$ is given to simulate the realistic distribution along the radial direction.

For the temperature distribution inside the furnace, the distribution along the wall $T_{W}$ is set to 973 K, while the distribution along the center-line is given as:

(11) $$T_{C}=\left(1-f_{a}\right)\left[B_{V}\frac{T_{ad}}{2}+B_{\mathrm{char}}\frac{T_{ad}}{2}+\left(2-B_{V}-B_{\mathrm{char}}\right)T_{in}\right]+f_{a}T_{exit},$$

where $f_{a}=z/L_{z}$ is the axial factor; $T_{ad}$, $T_{in}$, and $T_{exit}$ are the adiabatic temperature (2663 K), inlet temperature (600 K) and exit temperature (1523 K) of the medium, respectively; $B_{V}={\left(z/L_{z}\right)}^{0.2}$ and $B_{\mathrm{char}}=0.2{\left(z/L_{z}\right)}^{0.1}$ are the burnout ratios of volatiles and char, respectively. Taking $T_{W}$ and $T_{C}$ into Equation (10), the temperature distribution inside the furnace can be obtained.

For the mole fractions of gases inside the furnace, the distributions along the wall and center-line are given by:

(12) $$\begin{array}{l} \Phi_{W,mf}=B_{V}\Phi_{V,mf}+B_{\mathrm{char}}\Phi_{\mathrm{char},mf}+\Phi_{{in,mf}^{\prime }}\\ \Phi_{C,mf}=B_{\mathrm{char}}\left(\Phi_{\mathrm{char},mf}+\Phi_{V,mf}\right)+\Phi_{in,mf}. \end{array}$$

where $\Phi_{W,mf}$ and $\Phi_{\mathrm{char},mf}$ are the mole fractions produced by combustion of volatiles and char, respectively; $\Phi_{in,mf}$ is the mole faction entering the domain. Herein, CO_2_ and H_2_O are considered as the radiating gaseous species. In the air-based coal-fired furnace, $\Phi_{in,mf}=0$ for both CO_2_ and H_2_O; $\Phi_{V,mf}=0.0636$ and $\Phi_{\mathrm{char},mf}=0.0909$ for CO_2_; $\Phi_{V,mf}=0.0744$ and $\Phi_{\mathrm{char},mf}=0$ for H_2_O [37].

For the volume fraction of char and ash particle, under the assumption of the ash wrapped in the exterior of spherical char core and considering the diameter change of during combustion, the volume fraction of particle $f_{p}$ is given as [37]:

(13) $$D_{\mathrm{core}}=D_{\mathrm{core},in}{\left(1-B_{\mathrm{char}}\right)}^{\alpha },$$

(14) $$D_{p}=\sqrt[{3}]{D_{p,in}^{3}-D_{\mathrm{core},in}^{3}+D_{\mathrm{core}}^{3}},$$

(15) $$f_{p}=f_{p,in}\cdot \frac{D_{p}^{3}}{D_{p,in}^{3}},$$

where α is the structural parameter, $D_{\mathrm{core},in}$ and $D_{p,in}$ are the diameters of core and particle entering the domain, respectively, and *f_p,in_* is the volume fraction of particle entering the domain. $D_{\mathrm{core}}$ and $D_{p}$ are the diameters at the burnout ratio of $B_{\mathrm{char}}$, respectively. Herein, α=0.25, $D_{p,in}=60\;\mu m$, and $D_{\mathrm{core},in}^{}=0.45D_{p,in}$ [38]. For the soot, the volume fraction of soot in the furnace is set as uniformly distributed at 5 ppm.

**Figure 1:**
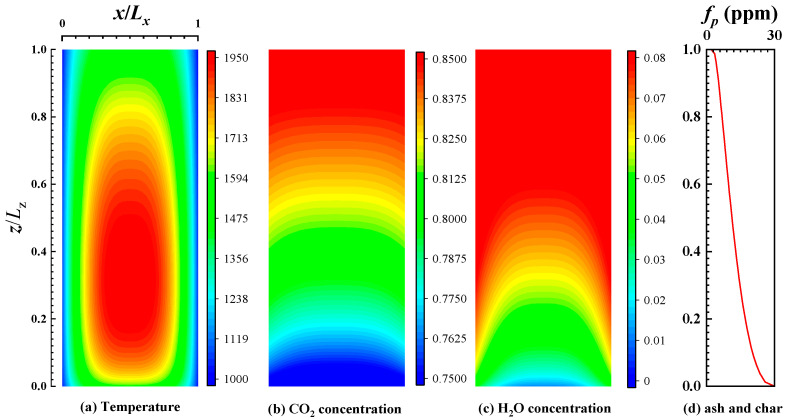
The distributions of temperature and species concentration: (**a**) temperature, (**b**) CO_2_, (**c**) H_2_O, (**d**) char and ash.

According to the simplified combustion model, distributions of temperature, CO_2_ concentration and H_2_O concentration in a two-dimensional cross section at the midline (*y* = 25 m) of the furnace are given in Figure 1a, Figure 1b, and Figure 1c, respectively. Meanwhile, the distribution of particle concentration with height is given in Figure 1d.

### 3.2. Spectral Emittance of Deposits

**Figure 2:**
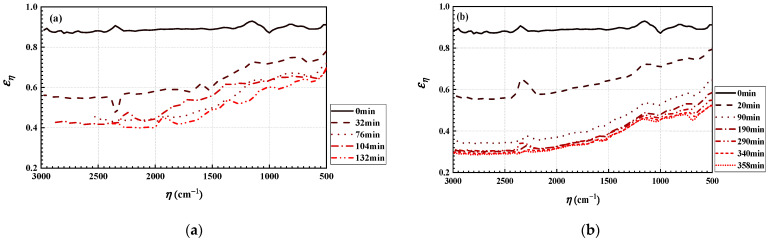
Spectral emittance of ash deposits as a function of the deposition time: (**a**) bituminous ash deposit; (**b**) sub-bituminous ash deposit.

Using an FTIR spectrometer, the spectral emittance of deposits left by burning bituminous and sub-bituminous coals were in situ measured between 3000 and 500 wavenumbers by Moore et al. [30]. As shown in Figure 2, both spectral emittance distributions of the bituminous and sub-bituminous ash deposits varied the deposition time. These measured history data of spectral emittance could be used to investigate the REG variation during the deposit growth. In the work, the bituminous coal sample was an Illinois #6 formation (represented by bituminous coal (Illinois #6 in the following text)), and the sub-bituminous coal sample was a Powder River Basin formation from Wyoming (represented by bituminous coal (Wyoming in the follow text)). The analytical results for the coal samples showed that the percentages of elements C, H, N, S and O were 57.97%, 4.27%, 1.08%, 3.33% and 8.85% in the bituminous coal (Illinois #6), respectively. The percentages of elements C, H, N, S and O were 71.45%, 6.02%, 1.1%, 0.17% and 21.26% in the sub-bituminous coal (Wyoming), respectively [32]. The component with the highest proportion in the two kinds of coal samples was SiO_2_. The proportions of SiO_2_ were 51.17% and 28.7% in the bituminous coal (Illinois #6) and sub-bituminous coal (Wyoming), respectively [32]. The detailed analysis results are given in Ref. [32]. Meanwhile, Fe_2_O_3_ content has a strong influence on the emittance of coal ash; higher Fe_2_O_3_ content in the bituminous coal leads to higher spectral emittance [39,40]. According to the analytical results, the proportion of Fe_2_O_3_ was 17.73% in the bituminous coal (Illinois #6) and 10.2% in the sub-bituminous coal (Wyoming). So, it is predictable that the emissivity of the bituminous coal (Illinois #6) is higher than that of the sub-bituminous coal (Wyoming).

## 4. Results

### 4.1. Distributions of the Local Heat Flux Density and the Local REG Rate on the Wall

**Figure 3:**
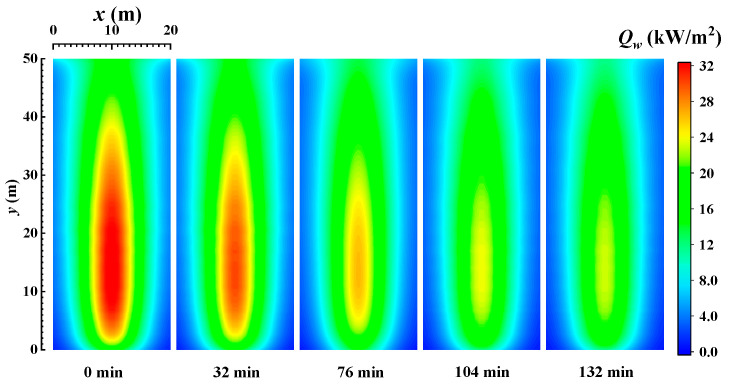
The distribution of the local heat flux density of the wall when burning bituminous coal (Illinois #6).

Figure 3 shows the distribution of the local heat flux density of the wall when burning bituminous coal (Illinois #6). From the figure, it can be seen that the heat flux density is higher near the centerline of the wall, and the highest heat flux on the wall is near the height of 15.7 m, which is mainly due to the highest temperature of the medium at this height and the strongest radiation heat transfer process. As the height continues to increase, the radiative heat flux gradually decreases due to the decreasing temperature of the medium, and there is a slight increase at the exit of the furnace. Because a furnace with large dimensions and high optical thickness is considered, the radiative heat transfer develops towards thermal diffusion [41], and the local wall heat flux density is related with the temperature gradient of the medium near the wall. Furthermore, as shown in Figure 1, in the region near the wall, the temperature gradient of the medium increases first and decreases with the height and has a maximum at 16.2 m. It is similar with the local heat flux density. As the burning time increases, the emissivity of the wall gradually decreases, and correspondingly, the heat flux density at the wall gradually decreases. As the burning time increases, the heat flux at the wall gradually stabilizes. The maximum value of heat flux density is reduced from 32.5 kW/m^2^ to 22.87 kW/m^2^ (decreasing 29.7%), and the total heat flux is reduced from 97.41 MW to 50.42 MW (decreasing 48.2%). It also indicates that the effect of ash deposits on the radiation heat transfer process in the furnace is very obvious, and reducing ash deposits can effectively improve the operation of the furnace.

**Figure 4:**
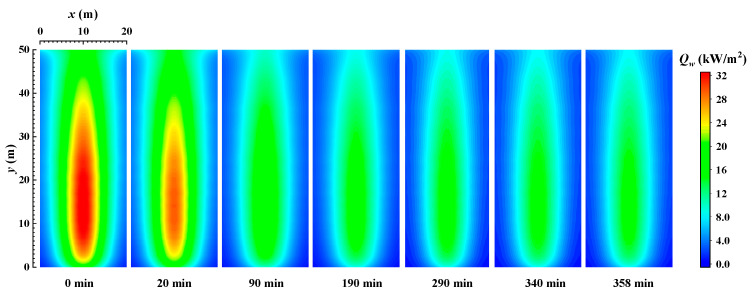
The distribution of the local heat flux density of the wall when burning sub-bituminous coal.

Figure 4 shows the distribution of the local heat flux density of the wall when burning sub-bituminous coal. Comparing the heat flux distribution when burning bituminous coal (Illinois #6) in Figure 3, we find that the difference in the type of coal does not significantly change the local heat flux distribution. The local heat flux density still has a peak near the centerline of the wall when sub-bituminous coal is burned. From Figure 2b and Figure 4, the heat flux decreases when the emissivity of the wall decreases with time, while the distribution of local heat flux on the wall gradually stabilizes when the combustion time is 190 min. Comparing the initial condition without ash deposits on the wall with the condition after 358 min, when the radiation on the wall and in the furnace stabilizes, the maximum value of heat flux density is reduced from 32.5 kW/m^2^ to 15.84 kW/m^2^ (decreasing 29.7%) and the total heat flux is reduced from 98.43 MW to 35.82 MW (decreasing 63.6%). Comparing the values of wall heat flux when burning bituminous coal (Illinois #6) and sub-bituminous (Wyoming), it can be seen that the ash deposits have a more serious weakening effect on the radiative heat transfer process when burning sub-bituminous coal (Wyoming), due to the lower emissivity of ash deposits on the wall and lower Fe_2_O_3_ content in the sub-bituminous coal (Wyoming).

**Figure 5:**
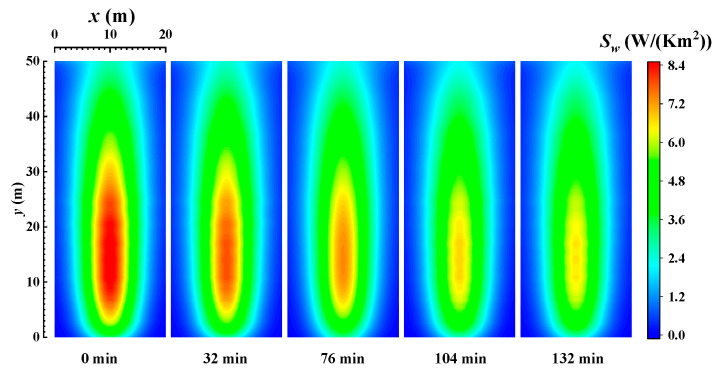
The distribution of the local REG rate on the wall when burning bituminous coal (Illinois #6).

Figure 5 shows the distribution of the local REG rate on the wall when burning bituminous coal (Illinois #6). From Figure 3 and Figure 5, it can be seen that the distribution of the local REG rate on the wall, similarly to the distribution of local radiative heat flux density on the wall, shows a trend of higher local REG rates near the centerline and lower local radiative entropy rates on both sides. The local REG rate reaches a maximum value at the centerline at a height of about 13 m. As the combustion time increases, the ash deposits on the wall gradually increase, and the emissivity of the wall gradually decreases (as shown in Figure 2). In addition, the radiative heat transfer process in the furnace is weakened, and the REG rate on the wall gradually decreases. Comparing the REG rate at the initial time without ash deposits and at the final time (132 min) when the radiative heat transfer process in the furnace reaches a steady state, the maximum value of the local REG rate on the wall decreases from 8.4 W/(Km^2^) to 6.2 W/(Km^2^). The reduction rate reaches 26.2%.

**Figure 6:**
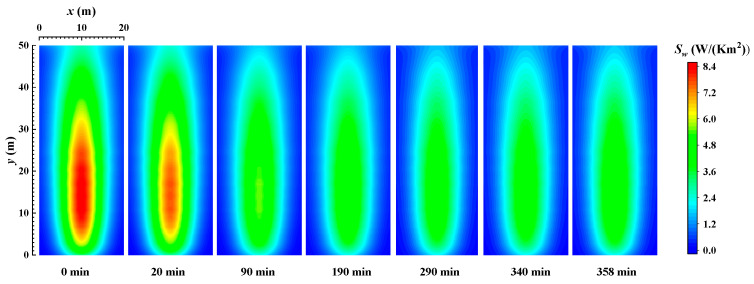
The distribution of the local REG rate on the wall when burning sub-bituminous coal (Wyoming).

Figure 6 shows distribution of the local REG rate on the wall when burning sub-bituminous coal. From the figure, it can be seen that the REG rate decreases with time due to the decrease in wall emissivity. The local REG rate on the wall gradually stabilizes when the combustion time is 190 min. Comparing the initial time of the wall without ash deposits and the final time when the radiation on the wall and in the furnace has stabilized, the peak value of the local radiation entropy generation rate on the wall decreases from 8.4 W/m^2^ to 5 W/m^2^, for a reduction rate of 35.7%. This indicates that the combustion process in a coal-fired boiler has a very significant impact on the irreversibility of radiative heat transfer on its walls. Additionally, comparing Figure 3 and Figure 5, and Figure 4 and Figure 6, it can be seen that the distribution of the local radiation entropy generation rate at the wall is very similar to the distribution of local heat flow density. The reason is that the enhancement of radiative heat transfer in the local region increases the thermodynamic irreversibility. Meanwhile, the decrease in REG when burning sub-bituminous coal (Wyoming) is also larger than that when burning bituminous coal (Illinois #6).

### 4.2. Total REG Rate

**Figure 7:**
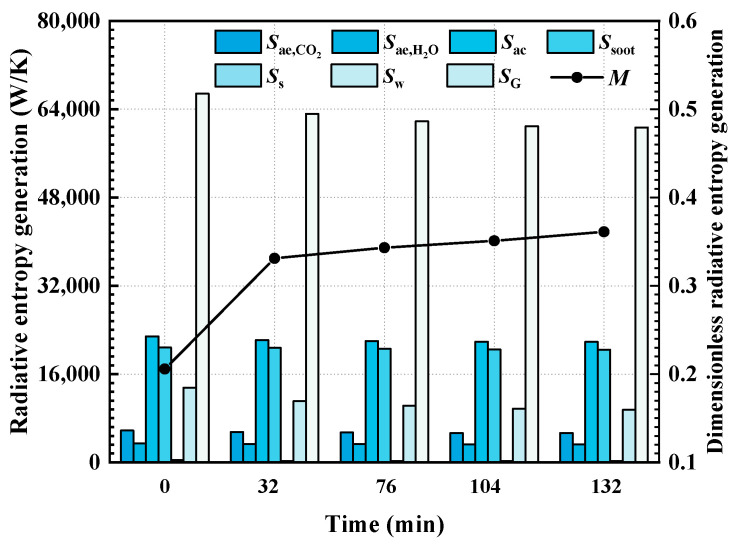
The total entropy generation rate and entropy generation rate due to various reasons during the combustion process of bituminous coal (Illinois #6).

**Figure 8:**
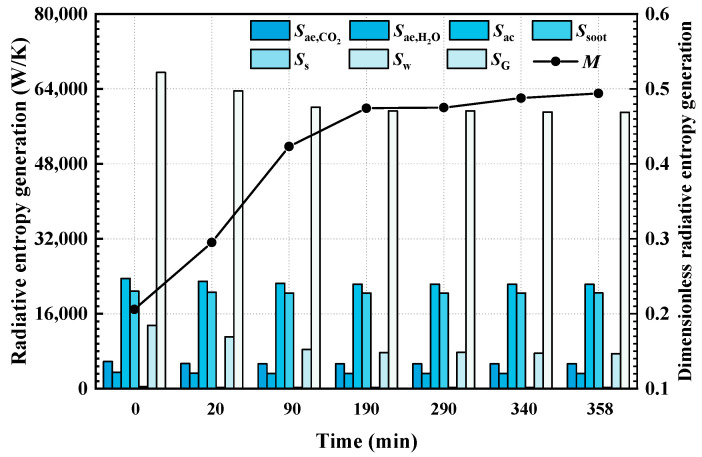
The total entropy generation rate and entropy generation rate due to various reasons during the combustion process of sub-bituminous coal (Wyoming).

Figure 7 and Figure 8 show the variations in total entropy generation rate and entropy generation rate due to various reasons (including gas radiation, soot radiation, particle radiation and wall radiation) during the combustion of bituminous coal (Illinois #6) and sub-bituminous coal (Wyoming), respectively. It can be found that the radiation entropy generation rate in the furnace is mainly due to the irreversibility of the absorption and emission processes of the particles in the furnace. Among the considered conditions, entropy generation caused by particle radiation is slightly higher than the entropy generation rate caused by soot radiation. Over time, among the radiation entropy generation rates due to various reasons, the most significantly affected is the entropy generation rate due to the radiation process on the furnace wall. The ash deposits directly affect radiation processes on the wall, which further leads to obvious change in the wall radiation REG. The radiative heat transfer process of the medium in the furnace is indirectly affected after the wall radiation change, so the effect of the ash deposits on REG in the medium is relatively lower. In the case of burning bituminous coal (Illinois #6), the total radiation entropy generation rate in the furnace at the initial time (no ash deposits on wall) is 66,825 W/K. As the burning time increases, the ash deposits on the walls increase, and when the burning time is 132 min, the entropy generation rate in the furnace decreases to 60,701 W/K (decreasing 9.1%). When burning sub-bituminous coal (Wyoming), the total entropy generation rate decreases from the initial time to 358 min and from 67,524 W/K to 59,007 W/K (decreasing 12.4%). Meanwhile, in terms of entropy generation number, it can also be found that the entropy generation number of the radiative heat transfer process in the furnace is also increasing with the increase in combustion time due to the ash deposits on the wall. It also indicates that the ash deposits on the wall not only lead to the weakening of the radiation heat transfer process in the furnace, but also cause a reduction in the efficiency of the radiative heat transfer process.

At the same time, comparing the REG rate and entropy generation numbers in the case of burning bituminous coal (Illinois #6) and sub-bituminous coal (Wyoming), it can be found that the difference in the total REG rate is not significant for the two kinds of coal. However, in the case of burning sub-bituminous coal (Wyoming), the entropy generation number of the radiative heat transfer process in the furnace is much higher than that of radiative heat transfer with bituminous coal (Illinois #6) combustion. According to the analytical results for the two types of coal, the Fe_2_O_3_ content of bituminous coal (Illinois #6) is higher than that of sub-bituminous coal (Wyoming), and the corresponding emissivity is higher. This leads to a further increase in REG and a decrease in entropy generation number. Therefore, the coal with higher Fe_2_O_3_ content is suggested to be selected in the operation of a furnace.

## 5. Conclusions

In this paper, we considered a 3-D high-temperature furnace filled with a gas-solid medium and analyzed the effect of ash deposits on the irreversibility and thermodynamic efficiency of radiative heat transfer in the furnace. Two types of coal were chosen, bituminous with high Fe_2_O_3_ content and sub-bituminous with low Fe_2_O_3_ content. Numerical results show that the radiation entropy generation rate in the furnace is mainly due to the irreversibility of the absorption and emission processes of the particles in the furnace, and the ash deposits have a significant effect on the irreversibility of the radiative heat transfer process on the wall. Meanwhile, when burning the two types of coals, the ash deposits in the furnace caused a decrease in the radiative heat flux rate on the wall and a decrease in the REG rate in the furnace. The ash deposits further cause an increase in the entropy generation number and a decrease in the thermodynamic efficiency of the radiative heat transfer process in the furnace. Meanwhile, the coal with high Fe_2_O_3_ content is suggested to be selected, as a high Fe_2_O_3_ content leads to high spectral emissivity and further increases the intensity and efficiency of radiative heat transfer.

## Data Availability

The authors confirm that all the data is contained within the article.

## References

[1] Cai J., Zheng H., Vardanyan M. (2023). Achieving carbon neutrality through green technological progress: Evidence from China. Energe Policy.

[2] Cartelle Barros J.J., Paz F.L., Coira L.M., de la Cruz López M.P., del Caño Gochi A., Soares A. (2022). New approach for assessing and optimizing the environmental performance of multinational electricity sectors: A European case study. Energe Conver. Manag..

[3] Jia Z., Lin B. (2021). How to achieve the first step of the carbon-neutrality 2060 target in China: The coal substitution perspective. Energy.

[4] Som S.K., Datta A. (2008). Thermodynamic irreversibilities and exergy balance in combustion processes. Prog. Energe Combust..

[5] Moran M.J., Shapiro H.N., Boettner D.D., Bailey M.B. (2018). Fundamentals of Engineering Thermodynamics.

[6] Agudelo A., Cortés C. (2010). Thermal radiation and the second law. Energy.

[7] Caldas M., Semião V. (2005). Entropy generation through radiative transfer in participating media. J. Quant. Spectrosc. Radiat. Transf..

[8] Zhang Z.M., Basu S. (2007). Entropy flow and generation in radiative transfer between surfaces. Int. J. Heat Mass Transf..

[9] Liu L.H., Chu S.X. (2007). Verification of numerical simulation method for entropy generation of radiation heat transfer in semitransparent medium. J. Quant. Spectrosc. Radiat. Transf..

[10] Sadeghi P., Safavinejad A. (2017). Radiative entropy generation in a gray absorbing, emitting, and scattering planar medium at radiative equilibrium. J. Quant. Spectrosc. Radiat. Transf..

[11] Zhang Z., Li Z., Lou C. (2019). Numerical analysis of radiative entropy generation in a parallel plate system with non-uniform temperature distribution participation medium. J. Quant. Spectros. Radiat. Transf..

[12] Shan S.Q., Zhou Z.J. (2019). Second Law Analysis of spectral radiative transfer and calculation in one-dimensional furnace cases. Entropy.

[13] Makhanlall D. (2013). Thermodynamic second-law analysis of radiative heat transfer in two-phase (particulate) media. J. Thermophys. Heat Transf..

[14] Bahrami A., Safavinejad A., Amiri H. (2019). Spectral radiative entropy generation in a non-gray planar participating medium including H_2_O and CO_2_. J. Quant. Spectrosc. Radiat. Transf..

[15] Zhang Z., Lou C., Li Z., Long Y. (2020). Evaluation of radiative entropy generation in a high temperature system including H_2_O, CO_2_ and soot with non-gray wall. J. Quant. Spectros. Radiat. Transf..

[16] Zhang Z.N., Lou C., Long Y., Benjamin M.K. (2021). Thermodynamics second-law analysis of hydrocarbon diffusion flames: Effects of soot and temperature. Combust. Flame.

[17] Yan H., Tang G., Wang C., Li L., Zhou Y., Zhang Z., Lou C. (2022). Thermodynamics irreversibilities analysis of oxy-fuel diffusion flames: Effect of oxygen concentration. Entropy.

[18] Sun H., Zhang Z., Sun H., Yao B., Lou C. (2022). Numerical investigation of exergy loss of ammonia addition in hydrocarbon diffusion flames. Entropy.

[19] Makhanlall D., Munda J.L., Jiang P. (2013). Radiation energy devaluation in diffusion combusting flow of natural gas. Energy.

[20] Rajabi V., Amani E. (2019). A computational study of swirl number effects on entropy generation in gas turbine combustors. Heat Transf. Eng..

[21] Lou C., Zhang Z. (2019). Experimental and numerical analysis of radiative entropy generation in industrial and boiler furnaces. J. Quant. Spectros. Radiat. Transf..

[22] Helble J.J., Srinivasachar S., Boni A.A. (1990). Factors influencing the transformation of minerals during pulverized coal combustion. Prog. Energy Combust. Sci..

[23] Richter W., Payne R., Heap M.P. (1986). Influence of Thermal Properties of Wall Deposits on Performance of Pulverized Fuel Fired Boiler Combustion Chambers. Mineral Matter and Ash in Coal ACS Symposium Series.

[24] Wall T.F., Bhattacharya S.P., Zhang D.K., Gupta R.P., He X. (1993). The properties and thermal effects of ash deposits in coal-fired furnaces. Prog. Energy Combust. Sci..

[25] Syrodoy S.V., Kuznetsov G.V., Gutareva N.Y., Salomato V.V. (2018). The efficiency of heat transfer through the ash deposits on the heat exchange surfaces by burning coal and coal-water fuels. J. Energy Inst..

[26] Melanie G., Elmar P., Primpuna H.S., David D. (2017). Impact of Solid Body Emissivity on Radiative Heat Transfer Efficiency in Furnaces—A Numerical Study. Energy Procedia.

[27] Vangaever S., Van Thielen J., Hood J., Olver J., Honnerovà P., Heynderickx G.J., Van Geem K.M. (2021). The effect of refractory wall emissivity on the energy efficiency of a gas-fired steam cracking pilot unit. Materials.

[28] Zhang Z., Lou C. Effect of wall spectral emissivity on heat flux and radiative entropy generation in three-dimensional enclosures. Proceedings of the 15th International Conference on Heat Transfer, Fluid Mechanics and Thermodynamics.

[29] Markham J.R., Best P.E., Solomon P.R., Yu Z.Z. (1992). Measurement of radiative properties of ash and slag by FT-IR emission and reflection spectroscopy. J. Heat Transfer. ASME.

[30] BaxterL L., Richards G.H., Ottesen D.K., Harb J.N. (1993). In situ, real-time characterization of coal ash deposits using Fourier transform infrared emission spectroscopy. Energy Fuels.

[31] Moore J.T., Cundick D.P., Jones M.R., Tree D.R., Maynes R.D., Baxter L.L. (2011). In situ measurements of the spectral emittance of coal ash deposits. J. Quant. Spectrosc. Radiat. Transf..

[32] El-Kolaly M.A., El-Agramy A.M., Hafez H.S. (1996). Effect of different activators on thermoluminescence properties of gamma irradiated cement clinker and its ferrite phase. Radiat. Eff. Defects Solids.

[33] Mirković M., Kljajević K., Nenadović S. (2021). Fly ash as a raw material for low-carbon cement clinkers and its radiological properties. J. Radioanal. Nucl. Chem..

[34] El-Hosiny F.I., El-Faramawy N.A. (2000). Shielding of gamma radiation by hydrated Portland cement lead pastes. Radiat. Meas..

[35] Modest M.F., Mazumder S. (2021). Radiative Heat Transfer.

[36] Liu F., Smallwood G.J., Gulder O.L. (2000). Application of the statistical narrow-band correlated-k method to low-resolution spectral intensity and radiative heat transfer calculations-effects of the quadrature scheme. Int. J. Heat Mass Transf..

[37] Kez V., Consalvi J.L., Liu F., Strohle J., Epple B. (2017). Assessment of several gas radiation models for radiative heat transfer calculations in a three-dimensional oxy-fuel furnace under coal-fired conditions. Int. J. Therm. Sci..

[38] Guo J., Hu F., Luo W., Li P., Liu Z. (2019). A full spectrum k-distribution based non-gray radiative property model for unburnt char. Proc. Combust. Inst..

[39] Bohnes S., Scherer V., Linka S. (2005). Spectral emissivity measurements of single mineral phases and ash deposits. Heat Transfer..

[40] Baxter L.L., Fletcher T.H., Ottesen D.K. (1988). Spectral emittance measurements of coal particles. Energy Fuels.

[41] Liu L.H., Chu S.X. (2006). On the entropy generation formula of radiation heat transfer processes. J. Heat Transfer..

